# Peripheral Visual Cues Contribute to the Perception of Object Movement During Self-Movement

**DOI:** 10.1177/2041669517736072

**Published:** 2017-11-21

**Authors:** Cassandra Rogers, Simon K. Rushton, Paul A. Warren

**Affiliations:** School of Psychology, Cardiff University, Cardiff, UK; Division of Neuroscience and Experimental Psychology, School of Biological Sciences, Faculty of Biology, Medicine and Health, University of Manchester, Manchester Academic Health Science Centre, Manchester, UK

**Keywords:** flow-parsing, flow-parsing, optic flow, peripheral vision, scene perception, motion, self-movement, object movement

## Abstract

Safe movement through the environment requires us to monitor our surroundings for moving objects or people. However, identification of moving objects in the scene is complicated by self-movement, which adds motion across the retina. To identify world-relative object movement, the brain thus has to ‘compensate for’ or ‘parse out’ the components of retinal motion that are due to self-movement. We have previously demonstrated that retinal cues arising from central vision contribute to solving this problem. Here, we investigate the contribution of peripheral vision, commonly thought to provide strong cues to self-movement. Stationary participants viewed a large field of view display, with radial flow patterns presented in the periphery, and judged the trajectory of a centrally presented probe. Across two experiments, we demonstrate and quantify the contribution of peripheral optic flow to flow parsing during forward and backward movement.

## Introduction

**Figure 1.**
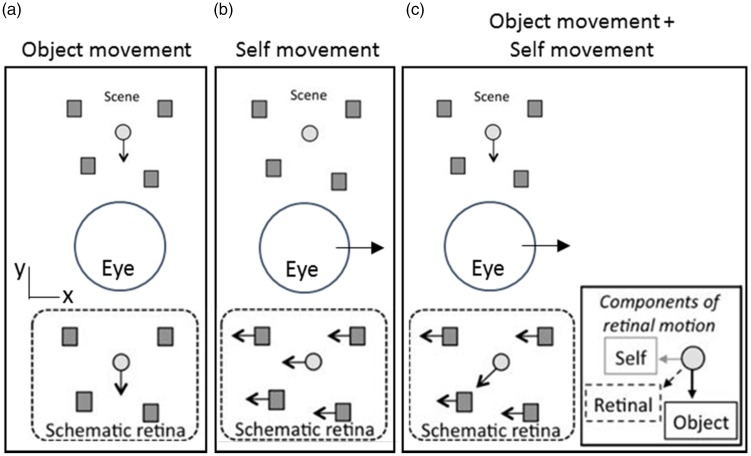
Retinal motion associated with stationary objects (dark grey) and a moving object (light grey) in the scene, whilst stationary (a) or during rightward self-movement (b and c). (Adapted from Warren, Rushton, & Foulkes, 2012.)

The ability to detect and estimate the movement of objects in the surrounding environment is vital. Without this ability, dangerous activities such as crossing the road would be very difficult. For a stationary observer, the visual detection of a moving object in an otherwise stationary scene is straightforward; moving objects in the world are indicated by motion in the image that is formed on the retina (retinal motion; Figure 1(a)). However, during self-movement, this task becomes more complex; objects that are stationary in the scene can be moving within the retinal image (Figure 1(b)) and the physical trajectories of objects that are moving in the scene are obscured (Figure 1(c)). Thus, during self-movement, the brain must decompose a complex pattern of retinal motion (Figure 1(c), insert) to separate components of retinal motion that are due to objects moving in the scene from components of retinal motion due to self-movement.

A general class of compensation solutions for this problem rests upon the brain generating a prediction of the sensory signals that should be expected from self-movement and then comparing this prediction to the actual incoming sensory data (von Holst & Mittlestadt, 1954). In practice, this process can be thought of as subtracting the predicted from the experienced sensory consequences of the movement. Given an accurate prediction, any remaining signal after the subtraction must then be due to the motion of other objects in the scene.

To be able to generate a prediction of expected sensory input, it is clear that information about current self-movement is required. This could come from either retinal (visual) or extra-retinal (non-visual) sources or a combination of both. Early investigations of this problem focused on extra-retinal information about self-movement (von Holst & Mittlestadt, 1954). Wallach (1987) and Gogel (1990) found that extra-retinal information about self-movement plays an important role in judgements of object movement. More recent work by Wexler (van Boxtel, Wexler, & Droulez, 2003; Wexler, Lamouret, & Droulez, 2001; Wexler, Panerai, Lamouret, & Droulez, 2001), Angelaki, Gu, and DeAngelis (2011; MacNeilage, Zhang, DeAngelis, & Angelaki, 2012) and Glennerster (Tcheang, Gilson, & Glennerster, 2005) has extended our understanding of these extra-retinal processes.

Over the last decade, research has begun to focus on how retinal information might also be used to enable compensation for the consequences of self-movement (Calabro, Soto-Faraco & Vaina, 2011; Calabro & Vaina, 2011; Fajen & Matthis, 2011; Matsumiya & Ando, 2009; Royden & Connors, 2010; Rushton, Bradshaw & Warren, 2007; Rushton & Warren, 2005; Warren & Rushton 2007; Warren & Rushton, 2008; Warren & Rushton, 2009; Royden & Moore, 2012; Warren, Rushton, & Foulkes, 2012; Fajen & Matthis, 2013; Fajen, Parade, & Matthis, 2013; Foulkes, Rushton, & Warren, 2013a, b; Royden & Holloway, 2014). When an observer moves, a characteristic, global, structured pattern of retinal motion arises which contains information about self-movement (Gibson, 1950). It should be possible to use such information to underpin a compensation solution.

The flow parsing hypothesis (Rushton & Warren, 2005) suggests that the brain uses incoming retinal motion to generate a direct prediction of current self-movement and then does something akin to a global subtraction (or filtering) of the (predicted) optic flow across the visual field. Considerable data have now been provided which is compatible with the use of such a visually based process (Rushton & Warren 2005; Rushton et al., 2007; Warren & Rushton, 2007; Warren & Rushton, 2009; Matsumiya & Ando, 2009; Royden & Connors, 2010; Calabro, Soto-Faraco & Vaina, 2011; Calabro & Vaina, 2011; Fajan & Matthis, 2011; Royden & Moore, 2012; Foulkes et al., 2013a, b; Fajen & Matthis, 2013; Fajen et al., 2013; Royden & Holloway, 2014). For example, Warren and Rushton (2009) show that when the left hemifield of an expanding radial optic flow stimulus is presented together with a probe object in the right hemifield, the perceived probe trajectory is biased. The direction of the bias is consistent with subtraction of the (absent) right hemifield of optic flow. Furthermore, the bias increases in size as the probe moves further into the empty hemifield—a difficult result to explain with an alternative account given that it suggests more interaction between two stimuli as they move further apart. However, this surprising result is predicted by flow parsing; motion in a radial flow field generally increases in magnitude with eccentricity, meaning the component to be subtracted (and hence the effect on the probe) should also increase with eccentricity. Recent work has also focused on developing models of the neural processes underlying flow parsing (e.g., Layton & Fajen, 2016; Royden & Holloway 2014; Royden, Sannicandro, & Webber, 2015).

Work on flow parsing to date has presented optic flow stimuli in central vision. However, peripheral vision is also known to make powerful contributions to both the perception of self-movement (Brandt, Dichgans, and Koenig, 1973; Berthoz, Pavard, & Young, 1975; Habak, Casanova, & Faubert, 2002; Lepecq, Jouen, & Dubon, 1993) and its control (Lee & Aronson, 1974; Bardy, Warren & Kay, 1999; Erikson & von Hofsten, 2005; Stoffregen, 1985). Furthermore, peripheral flow has a potential role in resolving some of the ambiguities in visual information present in central vision. For example, assuming noise in the coding of motion signals, the retinal flow associated with lateral translation is similar to that generated during a horizontal gaze rotation. There is no similar ambiguity with peripheral flow because gaze rotation and lateral translation generate quite different patterns of retinal motion. Lateral translation produces a radial flow pattern on either side of the observer, a contracting field on one side and an expanding field on the other, whereas gaze rotation produces laminar flow on both sides. Here, we assess the contribution of peripheral visual information to flow parsing during simulated forward and backward movement. Although forward movement is likely to be more common in everyday activity, we also included a backward self-movement condition for comparison.

Several different definitions of what constitutes peripheral vision and what constitutes central vision have been used previously. For example, Osaka (1994, as cited in Berencsi, Ishihara, & Imanaka, 2005) assessed the distribution of cones and rods across the retina and defined the central 2° to 4° of vision as ‘central’ with everything beyond classed as peripheral vision. Trevarthen (1968) classified peripheral vision as beyond the central 4° to 5° of vision. However, the majority of experimental studies consider central vision to cover a much larger portion of the retina, typically extending up to 15° from fixation (a 30° region in the centre of vision, see Brandt et al., 1973; Paulus, Straube, & Brandt, 1984; Post, 1988). For the purpose of our research, we used the latter definition and defined central vision as up to 15° from fixation.

### Overview of Experiments

In two experiments, we assessed the impact of optic flow presented in the periphery on the perceived trajectory of a target probe in central vision. In Experiment 1, we manipulated the eccentricity of the peripheral visual flow and found evidence for an effect on target trajectory (and hence a peripheral contribution to flow parsing) for flow stimuli up to 41° from fixation. The contribution of peripheral vision to flow parsing was found to decrease approximately linearly as a function of retinal eccentricity. In Experiment 2, we examined how information from different locations across the peripheral retina is combined. Our data suggest that the information from the most central part of the peripheral retina makes the primary contribution to the combined effect.

## Experiment 1

Flow parsing involves the subtraction of global components of motion. If a global component of motion is subtracted then every object in the visual field should be affected. To recap, Warren and Rushton (2009) looked for evidence of the subtraction process by probing empty locations within the visual stimulus. In a key condition, they placed the probe dot opposite a hemifield of radial flow that had a focus of expansion at the point of fixation. They found that the perceived trajectory of the probe dot was influenced by the presence of the flow (and the change showed a dependence on the position of the relative to the focus of expansion). The difference between physical and perceived trajectory was consistent with a global flow subtraction process.

**Figure 2.**
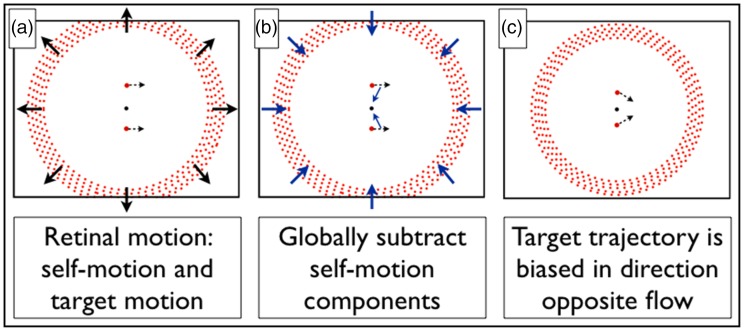
Schematic illustration of flow parsing with peripheral motion. Expanding flow condition depicted. (a) The retinal motion associated with forward self-movement, both possible target locations are indicated. The target moves across the screen left to right in a horizontal path. (b) Once a (radial) pattern of optic flow has been identified, a global subtraction process subtracts an expanding radial component of motion across the whole visual field (equivalent to adding a contracting radial component as illustrated here). (c) The perceived target trajectory once self-movement components have been parsed from retinal motion. The perceived target trajectory is biased inwards in this example and it would be biased outwards with contracting flow.

Warren and Rushton (2009) demonstrated the contribution of central vision to the parsing process. Here, we created a variant of Warren and Rushton (2009) to investigate if peripheral vision contributes, and if so, which parts of the periphery contribute. Expanding or contracting radial flow was presented at different eccentricities whilst a horizontally moving target appeared either above or below a central fixation point, as depicted in Figure 2.

If peripheral vision contributes to the flow parsing process, then the perceived trajectory of the target dot will be biased inwards in the presence of expanding radial flow (see Figure 2) or outwards in the case of contracting radial flow. By varying the eccentricity of the radial flow, we can assess the extent to which the effects of peripheral flow persist.

All targets moved across the screen from left to right, perceived trajectories are expressed in terms of a positive or a negative trajectory bias, where positive is anticlockwise (ACW; i.e., Figure 2(c), the target below fixation shows an ACW bias which would be coded as a positive relative tilt, and the target above fixation shows a clockwise [CW] bias, coded as a negative relative tilt). We summarise the results using the difference in relative tilt between the two target positions (see Supplementary Materials for detailed results at each flow eccentricity). We will look for evidence of a change in the relative tilt difference as a function of flow eccentricity. To do this, we conducted a two-factor ANOVA for each eccentricity condition with direction of radial flow (forwards or backwards) and target position (above or below) as factors. A significant contribution at each eccentricity would be indicated by a statistical interaction between target position and the direction of radial motion.

### Methods

#### Participants

Six postgraduate students (1 male) with an age range of 22 to 26 took part in the study and were naïve as to the experimental hypotheses. Participants were recruited from the School of Psychology, Cardiff University, and had normal or corrected-to-normal vision. Where vision was corrected, participants were asked to wear contact lenses. This additional restriction was put in place because the frames of a participant’s glasses might have obscured (at least partially) the peripheral stimuli used in the experiments.

All recruitment and experimental procedures reported herein adhered to the Declaration of Helsinki and were approved by the Ethics Committee of the School of Psychology, Cardiff University.

#### Apparatus

Computer-generated visual stimuli were presented on a large nonpolarising back projection screen using a ChristieT Digital Systems Projector (model DS+26). The spatial resolution of the display was 1400 × 1050 with a refresh rate of 60 Hz. The projected image size was 127 cm × 96 cm. When viewed at a distance of 40 cm, the projection area subtended 116° × 100° and had a resolution of 12 pixels/degree.

All experiments were coded in Pascal and OpenGL using Lazarus (an open source IDE), SDL v1.2 (https://www.libsdl.org) and the JEDI-SDL libraries (http://www.delphi-jedi.org). Stimuli were rendered on a computer running Windows XP with a NVIDIA Quadro NVS 420 Graphics card with four DVI outputs (driving two projectors and two peripheral monitors). All stimuli were drawn in red and presented on a black background unless otherwise stated. A red filter was placed in front of the projector to improve the contrast of the display. Anti-aliasing was set to 2 × multisample antialiasing in order to ensure smooth motion of the stimuli at such a close viewing distance. For trajectory judgements, participants used a physical ‘jog-wheel’ (a rotating dial) to orient an on-screen response line to match the trajectory they perceived.

#### Stimuli

Radial flow was presented on the projection screen and simulated forward or backward translation. The flow stimulus consisted of 3,500 red limited lifetime dots (1 second lifetime, 0.6° diameter). Dot motion was appropriate for an observer translating either forward or backward at a rate of 30 cm/s (a slow walking pace) through a volume of 5.33 m × 5.33 m × 10 m that was centred 133 cm in front of the observer. When a dot moved offscreen or had been present for more than 1 s, it was redrawn at a new on-screen location at the end of the volume, ahead of the observer. Dots were presented in an annulus (peripheral band). Dot lifetime was staggered so that dots disappeared and reappeared asynchronously. Approximately 600 dots were visible at any one time.

**Figure 3.**
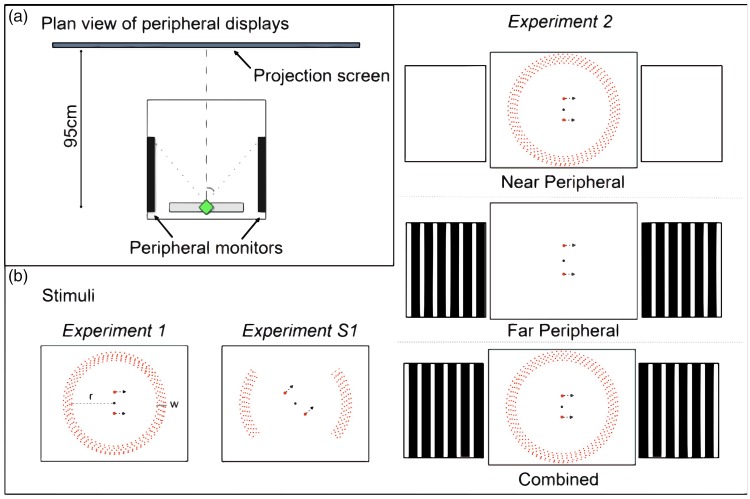
(a) Plan view of display devices for peripheral stimuli for Experiments 1 and 2. In Experiment 1, only the projection screen was used and the observer was seated, with a viewing distance of 40 cm. In Experiment 2, both the projection screen and the side screens were used and the viewing distance was 95 cm. The observer is seated with head in chinrest (green diamond) looking straight ahead. In Experiment 2, the Near peripheral stimulus (expanding or contracting radial flow) was presented on the projection screen and the Far peripheral stimulus (moving bars) was presented on peripheral monitors, each positioned with the nearest edge 45° from the (dashed) line of sight. (b) Schematic diagrams of stimuli used in experiments. Experiment 1 and Experiment S1 (control, see Supplementary Materials) used only the central projection screen to present flow, while Experiment 2 used the central projection screen and two peripheral monitors to present flow stimuli. Experiment 1 dashed line (*r*) shows inner annulus radius and solid line (*w*) indicates flow width.

Five inner annulus radii (*r*, see Figure 3(b)) were used, visual angles of 23°, 27°, 32°, 37° and 41° (16.8, 20, 25, 30, 35 cm in on-screen dimensions, as presented in results). The annulus width (*w*, see Figure 3(b)) was 14° at a radius of 23°. The annulus width increased with annulus radius, it was M-scaled for eccentricity using the method described by Rousselet, Husk, Bennett, and Sekuler (2005; see Supplementary Materials).

A central fixation dot was presented at the centre of the flow stimulus together with a small (0.3°) circular red target probe placed either above or below fixation at 4° eccentricity. Target motion was predominantly horizontal and rightwards (although see below) at 0.6°/s. All targets appeared to the left hand side of the midline and translated rightward along the specified trajectory. The target passed through the vertical midline of the screen half way along its travelled path.

#### Design

Three independent variables were manipulated: the direction of simulated self-movement (2 levels: expanding or contracting flow), the position of the target (2 levels: −4°/+4° from fixation) and the eccentricity of the peripheral flow (measured by the inner annulus radius [*r*], 5 levels: 23°, 27°, 32°, 37° and 41°).

To avoid potential response biases, rather than repeatedly presenting the same trajectory, the target had a fixed initial position and the trajectory varied within a range above and below horizontal. This manipulation prevented the observer from knowing whether perceived upwards motion was physical or due to flow parsing on any given trial. In each condition, there were nine physical target trajectories (spanning the range ± 32° from horizontal in 8° steps), resulting in a total of 180 trials (2 × 2 × 5 × 9), which were all completed in a single experimental session of approximately 45 min. The order of the trials was randomised and all eccentricity conditions were interleaved.

The dependent variable was relative tilt, which was calculated as the difference between the on-screen trajectory and perceived trajectory reported by the observer. A within-subjects design was used and data collection for each participant was completed in a single experimental session.

#### Procedure

Observers were seated on a static height-adjustable chair, in a dark room with their eyes level with the fixation point and approximately 40 cm viewing distance from the centre of the projection screen. It was not feasible to provide participants with a chin rest at this viewing distance but participants (who had all taken part in previous psychophysical experiments) were instructed to keep as still as possible during the experiment.

**Figure 4.**
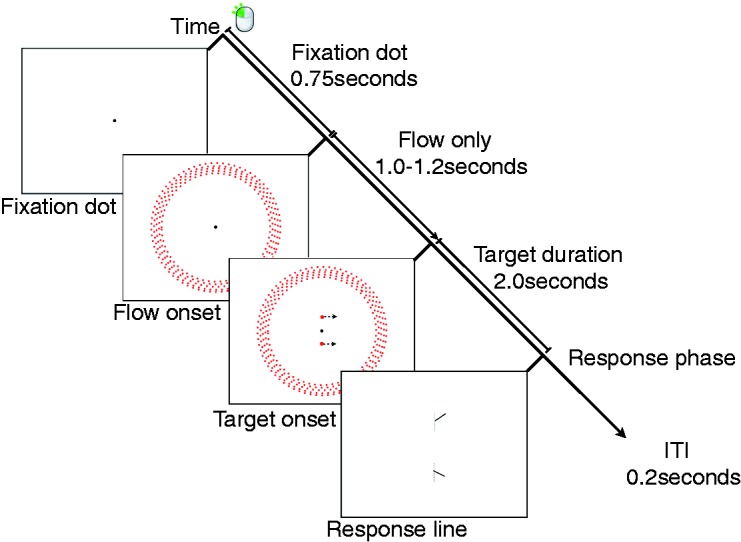
Timeline for Experiment 1. Both possible target locations are indicated but only one target was presented on each trial. The response line appeared in the same location as the target.

The timeline of each trial, depicted in Figure 4, was as follows: A fixation dot was presented. Participants maintained fixation on the central dot throughout each trial. After 0.75 s, peripheral flow appeared which simulated forward or backward translation at a speed of 30 cm/s. Following a variable 1 to 1.2 s delay, the target was presented above or below fixation and moved at constant speed of 0.6°/s (1 cm/s) for a 2-s duration. Immediately after the probe disappeared, a short 2D response line (∼3° in length) which rotated about the point at which the target initially appeared was presented with a randomised initial line orientation on each trial. Participants rotated the line using a rotating dial or ‘jog-wheel’ in order to indicate the perceived trajectory of the target. Participants were told that if they did not perceive the target to move along a straight path, then they should set the response line to match the mean linear trajectory of the target. Once the participant had set the on-screen line, they clicked a button on the jog-wheel to move on to the next trial. By delaying their click, observers could opt to take a break before continuing. Four enforced breaks of 15 s duration were imposed at regular intervals during the experiment.

#### Analysis

To equate relative tilts across the different target trajectories, a simple transformation was applied to the raw data^1^ (see Warren & Rushton, 2007, for further details):

$$\theta_{\text{i}}{=\text{tan}}^{-1}{(\text{sin}(\theta }_{\text{p}}{-\theta }_{\text{R}})/\text{sin}\theta_{\text{p}})$$

where the P and R subscripts refer to perceived and real on-screen trajectory angles. In the results that follow, $\theta_{\text{i}}$ is referred to as the relative tilt.

To present appropriate within-subject error bars in figures, the data were normalised according to the method described in Cousineau (2005). Variance due to individual differences was removed from the dataset by subtracting each participant’s average relative tilt from the same participant’s relative tilt in each experimental condition. Using the adjusted dataset, the standard error was then calculated for each condition.

Under this experimental design, flow parsing predicts that changing either the target position or flow movement direction should lead to reversals in the sign of the relative tilt in an unbiased observer. The combination of the two effects means that we should find a statistical interaction between target position and flow direction. Accordingly, we conducted a 2 × 2 repeated measures ANOVA at each flow eccentricity and looked for a simple interaction between the flow direction and target position as a marker of flow parsing (rather than insisting on a sign change). The presence of an interaction provides statistical support for a contribution to flow parsing at the eccentricity considered. Since a directional interaction was predicted, probability values from the ANOVA interaction term were adjusted (p/2; Wuensch, 2006), because the error term for the interaction in an ANOVA does not assume a directional effect. Note that the test for an interaction is robust in the case of observers who have a systematic tendency to perceive, or report, a trajectory inaccurately.

In addition to looking for evidence of flow parsing at each flow eccentricity, we looked to see if the magnitude of the flow parsing effect (relative tilt) increased (or decreased) as a function of the eccentricity of the radial flow. We conducted this analysis on the difference of the relative tilt between the above and below target positions, which essentially provides an index of the magnitude of the flow parsing effects observed across the two probe positions and has the added advantage of doubling the size of the effect. A 5 (flow eccentricity) × 2 (flow direction) within-subjects ANOVA was then performed. If flow parsing effects decreased as a function of flow eccentricity, then we would expect to see an interaction between these two factors.

### Results and Discussion

We found that the presence of expanding or contracting visual flow in peripheral vision biased the perceived trajectory of a central target. At each tested flow eccentricity and target position, perceived target trajectory was biased in the direction opposite to the flow—indicative of global subtraction and in line with a peripheral contribution to flow parsing.

**Figure 5.**
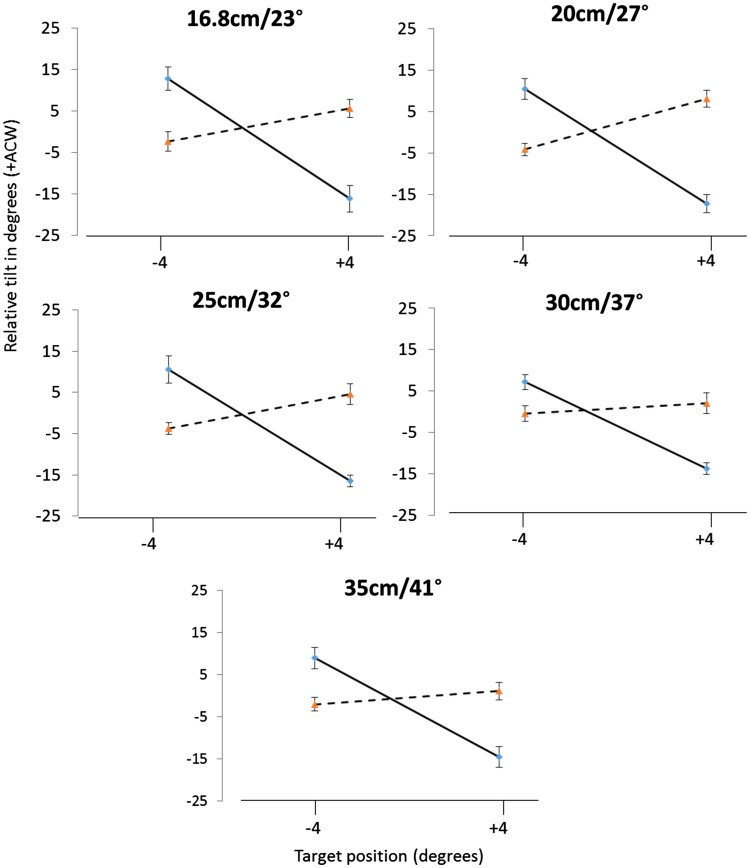
For each flow eccentricity: Relative tilt in degrees, shown in the vertical axis, as a function of flow direction (expanding flow—blue squares, Contracting flow—red triangles) and target position for above and below target locations, shown on the horizontal axis. Error bars show within-subjects SE.

For targets located below fixation (−4), target trajectory was ACW during expanding flow and CW during contracting flow. When the target was located above fixation (+4), we observed the opposite pattern with expanding flow biasing target trajectory in a CW direction and contracting flow resulting in an ACW bias. This pattern of results indicates a contribution of peripheral flow to a flow parsing mechanism and was statistically significant (interaction, all *p* < .01) at each flow eccentricity (Figure 5; see Supplementary Materials for full statistical results).

**Figure 6.**
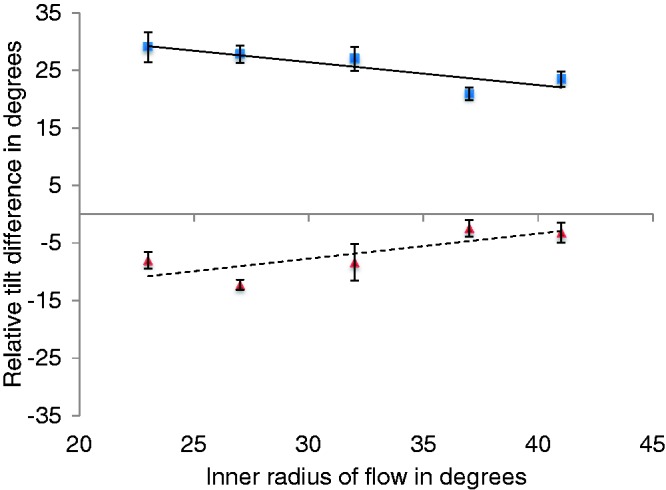
Relative tilt difference in degrees (i.e., the difference between relative tilts when the probe was positioned above and below the fixation point) as a function of flow direction (expanding flow—blue squares, contracting flow—red triangles) and flow eccentricity, shown on the horizontal axis. Error bars show within-subjects SE.

There was also a significant interaction between flow eccentricity and flow direction, *F*(4, 20) = 7.891, *p* < .001, the magnitude of the effect differed as a function of the direction of the flow and the eccentricity of the flow. This interaction which is evident in Figure 6 indicates that relative tilt difference decreases with increasing flow eccentricity.

We also found that relative tilt effects were larger in the expanding flow conditions (Figure 6). The magnitude of the difference in relative tilt between the two target positions was significantly larger for expanding flow than contracting flow, *F*(1, 5) = 265.332, *p* < .001. The marked difference between expanding and contracting flow is evident in this figure. This difference might indicate a response bias, and the similar gradient of the two lines is compatible with this. However, asymmetries between expanding and contracting flow have been reported before (e.g., Edwards & Ibbotson, 2007, showed faster onset and larger magnitude of vection in response to contracting flow than expanding flow but there is some debate in this area and other studies have demonstrated a greater response to expanding flow, i.e., Reinhardt-Rutland, 1982). The differences observed in this experiment between expanding and contracting flow might be due to broader differences in the neural processing of expanding and contracting motion. As forward self-movement is typically faster than backward self-movement, there may well be differences in the speed tuning of neurons in relation to these two directions of motion. The second control experiment (Experiment S2) provides some data on the sensitivity to motion between forward and backward self-movement using the present flow stimuli. The results of S2 did not indicate any differences in sensitivity.

## Experiment 2

The results of Experiment 1 show that peripheral flow contributes to flow parsing but that the contribution decreases as the eccentricity of flow increases. However, we were only able to explore the contribution over a limited range of eccentricities. In Experiment 2, we explore further into the periphery and examine how information from different parts of the peripheral retina is combined. To do this, we defined near (15°–45° from fixation) and far (45°–135° from fixation) peripheral regions. To stimulate far periphery, we placed monitors to either side of the participant’s head. This setup was inspired by the peripheral vection experiments in Lepecq et al. (1993) which used moving striped vertical bar stimuli presented on similarly arranged screens. This experimental setup was reported to create a compelling sense of vection and therefore should provide a strong visual cue to self-movement in this experiment.

Target movement parameters and the relative tilt measure of perceived target trajectory were the same as in Experiment 1. As in Experiment 1, the contribution of peripheral motion to flow parsing would be indicated by a significant interaction between flow direction and target position.

### Methods

#### Participants

Five undergraduate students (2 male) with an age range of 18 to 21 were recruited using an online participant panel and received course credit. The same eligibility restrictions and ethical procedures as for Experiment 1 were applied. All participants were naïve to the experimental hypotheses.

#### Apparatus

The central projection screen apparatus was the same as that used in Experiment 1, but we used an increased viewing distance of 95 cm. At this distance, the projected image size was 67.5° × 53.6° and there were 21 pixels/degree. In addition, two 19″ (aspect ratio 5:4) BENQ LCD monitors (model number: Q9T4) with a resolution of 1280 × 1050 were placed either side of the observer’s head, in a portrait orientation (see Figure 3(a)). The monitors faced each other and were separated by a total distance of 43 cm with the chinrest for observers centred between the two monitors. Thus, each monitor was approximately 15 cm from the observer’s nearest eye. Each monitor subtended approximately 90° horizontally and 100° vertically. For an observer seated with their head in the chin rest and looking straight ahead, the front edge of each peripheral display (monitor screen) was 45° from the (cyclopean) line of sight (see Figure 3). The monitors at the side of the head were covered with a red lighting gel to increase the contrast of the stimuli and reduce ambient light which might have otherwise increased the saliency of the edges of the monitors. Note that the arrangement of the apparatus meant that there were gaps between the vertical edges of the central and peripheral screens.

#### Stimuli

As depicted in Figure 3 (right hand side), three peripheral flow conditions (Near, Far and Combined) were employed. The Near peripheral flow was generated using the same method as reported for Experiment 1 and had an inner radius (*r*) of 40 cm (22.8°) and an outer radius of 50 cm (27.8°) which was a similar eccentricity to inner edge of the least peripheral stimuli presented in Experiment 1 (inner radius [*r*] of 23°). Far peripheral flow was presented on monitors either side of the head. Stripes were used in the periphery similar to Lepecq et al.’s (1993) stimuli and because pilot testing demonstrated this stimulus gave a more compelling sense of self-movement than the limited lifetime dots that were used in central vision. Vertical stripes were positioned at a virtual distance of 50 cm from the observer’s nose on two parallel planes, either side of the head and orthogonal to the fronto-parallel plane and moved parallel to the line of sight away from the central projection screen. The Combined flow condition presented the Near and Far peripheral stimuli simultaneously.

As before, the target was a small circular dot (diameter: 0.12°) positioned either above or below fixation at an eccentricity of 4° from fixation and moved rightwards at a speed of 0.6°/s. The diameter of the flow dots, target size, fixation dot size and target position were all scaled in accordance with the increased viewing distance (95 cm vs. 40 cm).

#### Design

We manipulated three variables in this experiment: target position (−4/+4°), flow direction (expanding/contracting) and peripheral region (Near/Far/Combined). For each of the 12 resulting conditions, the nine tilt trajectories we employed in the previous experiment were presented twice. Near, Far and Combined conditions were presented in separate blocks consisting of 72 trials each. As before, we measured the relative tilt in degrees. All participants saw all conditions and the order of peripheral conditions was counterbalanced across participants. Each participant completed the three peripheral conditions in a single experimental session lasting approximately 45 min.

#### Procedure

Participants were seated with their head in a chinrest and instructed to maintain fixation on the dot in the centre of the screen during stimulus presentation. The trial procedure timings were identical to Experiment 1 (see Figure 4) and the Near, Far or Combined peripheral flow conditions were presented in separate experimental blocks. A 15-s enforced break was included halfway through each block. Because there were more trials in Experiment 2 than Experiment 1, in order to reduce eyestrain and minimise fatigue, the ceiling lights were turned on and participants had a short (∼2 min) break between each peripheral condition (Near/Far/Combined).

#### Analysis

As before, to assess whether peripheral flow contributes to flow parsing, we conducted a 2 (expanding/contracting flow) × 2 (−4/+4 target position) repeated measures ANOVA on the data from each peripheral condition.

To investigate how information from Near and Far periphery is combined, we compared the magnitude of the effect in the Combined condition to the sum of the magnitude of Near and Far effects. Specifically, we conducted a regression analysis to evaluate how well the Combined condition data could be predicted from the linear sum of near and far data.

### Results and Discussion

**Figure 7.**
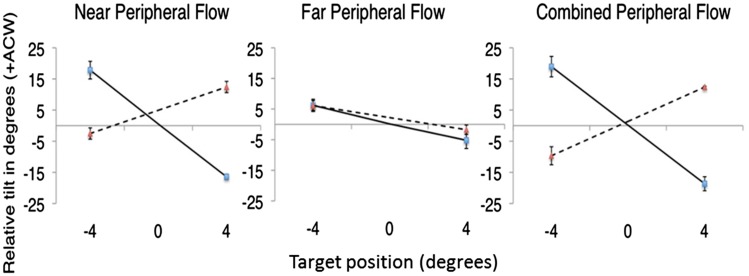
Relative tilt as a function of target position for each peripheral condition (solid lines—expanding flow, broken lines—contracting flow). Error bars show within-subjects SE.

The results corroborated the findings of Experiment 1, they showed a peripheral contribution to flow parsing when flow was presented in near peripheral vision (Figure 7, left panel). The interaction between flow direction and target position was highly significant, *F*(1, 4) = 185.351, *p* < .001. In the Far peripheral condition (central panel, Figure 7), the magnitude of relative tilt observed was much lower than in the Near condition. Additionally, the effect of Flow direction and target position upon relative tilt was less pronounced, but a significant interaction was still present, *F*(1, 4) = 6.544, *p* = .0315. The data in the Combined condition (Figure 7, right panel) looked rather similar to the Near condition and once again a significant interaction between flow direction and target position was observed, *F*(1, 4) = 68.249, *p* = .005.

For each flow direction (expanding or contracting), a linear regression analyses was conducted to test the extent to which the relative tilt data in the Combined condition could be predicted by the linear sum (Near + Far) data. For the expanding flow condition, statistical testing showed the linear sum model accounted for 97% of the variance in the Combined data, *β* = 0.788, *R*^2 ^= .971, *F*(1, 8) = 269.838, *p* < .001. For Contracting flow, the linear sum model accounted for only 34% of the variance in the Combined data, *β* = 0.817, *R*^2 ^= .343, *F*(1, 8) = 4.170, *p* = .075, *n.s*. Taken together, these results suggest that the contribution of far peripheral flow to flow parsing is limited. There is some indication that the linear sum model can account for the data in the expanding but not the contracting flow conditions. Rather than suggesting that the brain does not combine information over the retina, it is likely that the result in the Contracting condition is due to the small magnitude of the effect of far peripheral flow on perceived probe trajectory.

### Contribution of Far periphery

Given the linear relationship between eccentricity and the magnitude of the flow parsing effect in Experiment 1, we can attempt to predict the amount of relative tilt that is expected for the Far peripheral condition. Before doing so, however, we note an important caveat: Image speed depends on both distance from the observer and self-movement speed. The ability to factor out the effect of distance depends on the quality of the distance cues available. Distance cues are typically plentiful in central vision but drop off in peripheral vision. Consequently, for the far peripheral stimuli, the magnitude of any flow parsing is probably based on raw image speed alone. Had we placed the virtual bars closer, the image speed would have been higher; had we placed them further, then the image speeds would have been lower. Therefore, the magnitude of the flow parsing effect we observe with far periphery alone is relatively arbitrary. With that caveat clearly stated, we looked at the observed magnitude of the flow parsing effect in far periphery and the magnitude predicted from an extrapolation of the results observed for central and near peripheral regions in Experiment 1.

**Figure 8.**
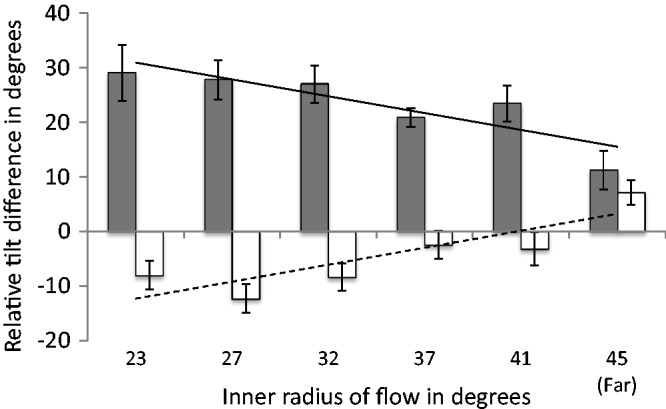
Composite data from all conditions of Experiment 1 and Far condition from Experiment 2. Solid and dashed lines show the predicted relative tilt difference for flow at 45° eccentricity on the basis of Experiment 1.

The nearest edge of the far peripheral stimulus was 45° from fixation and the flow was presented only on the left and right of the head. We fitted a regression model to the data from Experiment 1 and used the model to predict relative tilt difference for a case where flow was presented at 45° eccentricity. The composite data and predictions are shown in Figure 8.

The prediction on the basis of the data from Experiment 1 for flow presented at 45° eccentricity is broadly in line with the data collected from the Far condition of Experiment 2. In the following section, we consider some additional factors that could impact on the flow parsing effects observed.

### Further Influences on the Magnitude of the Flow-Parsing Effect, Supplemental Experiments

In further supplementary experiments, we investigated the effect of further stimulus parameters. In Supplementary Experiment S1, we investigated whether reducing the area of the flow, specifically whether presenting flow only in two quadrants (as in Experiment 2), impacted upon the magnitude of flow parsing. The results of the experiment indicated that the presence of flow stimuli on only the left and right quadrants did lead to a reduction in relative tilt difference compared with when stimuli were presented in all four quadrants of the visual field. Specifically, we found that the relative tilt for expanding flow reduced by 34% and for contracting flow by 49%. Nonetheless, even with the removal of half of the area of the visual field (the above and below quadrants), the overall pattern of results was similar in this supplementary experiment.

In supplementary Experiment S2, we investigated the impact of stimulus type and peripheral position between Experiments 1 and 2 (near peripheral dots vs. far peripheral stripes), whether the two stimuli provided equally good motion signals. We did this by comparing speed discrimination thresholds. We found no effects of stimulus type on speed discrimination.

In sum, across Experiments 1 and 2, we find that peripheral flow does contribute to flow parsing but the contribution decreases with eccentricity. This reduction appears to follow a linear function. We find no compelling evidence that flow presented beyond 40° eccentricity makes a significant contribution to flow parsing.

## Discussion

We set out to investigate whether optic flow presented in the periphery could contribute to the flow parsing process during simulated forward and backward movements of the observer. The experiments reported here demonstrate that peripheral visual flow can contribute to flow parsing. When self-movement information is presented in peripheral vision, it leads to a systematic effect on perceived object trajectory in line with the flow parsing account. Experiment 1 showed that the magnitude of the effect on perceived object trajectory decreases as peripheral flow becomes more eccentric. Experiment 2 compared the contributions of flow in the Near and Far periphery and how these contributions might be combined. The results extended the findings of the first experiment, and again showed that the contribution of peripheral flow to flow parsing decreases with increasing retinal eccentricity. We examined how flow from different portions of the periphery was combined. We found some evidence for combination across regions; however, the fact that we found greatly reduced effects when flow was in far periphery makes it hard to draw a strong conclusion from these data.

In our experiments, we ask observers to maintain fixation at the focus of the flow field. Studies have shown that this is the location that observers typically fixate (Land & Lee, 1994; Lappe, Pekel, & Hoffmann, 1998, 1999; Mourant, Rockwell, & Rackoff, 1969; Niemann, Lappe, Büscher, & Hoffmann, 1999). Because observers are looking at the focus of the flow field, retinal eccentricity and eccentricity defined relative to the focus of the flow field are equivalent. Consequently, we cannot distinguish between the two. If an observer fixates a point away from the focus, the difference between eccentricity relative to the fovea and eccentricity relative to the flow field becomes important. Research has shown that ability to judge direction of heading is relatively independent of the eccentricity of the focus of expansion (Crowell & Banks, 1993). Therefore, we would expect little difference in the flow-parsing process. However, further experimental work is needed to confirm this prediction.

We now consider potential explanations for the decreased contribution to flow parsing of peripheral retina. Flow parsing relies on an accurate estimate of self-movement direction. In addition, retinal image speed is a function of both the speed at which the observer is translating and the distance of the scene objects from the observer so the availability of these cues is important for flow parsing. In central vision, the availability of binocularly overlapping visual fields and high acuity allow for precise estimates of the distance that is necessary for accurate estimation of observer translation speed. As the eccentricity increases, raw image speed persists but the available information about distance decreases, so consequently, the precision of estimates of self-movement speed will also decrease. The decrease in the precision of estimates of self-movement direction or self-movement speed should lead to the down-weighting of flow with eccentricity in the flow parsing process.

The research reported here provides a foundation for exploring the potential contribution of peripheral vision to flow parsing for other forms of self-movement. The case of lateral self-movement will be of particular interest as the peripheral flow structure unambiguously specifies that the observer is undergoing lateral translation. This differs from central vision where the flow due to lateral translation is difficult to distinguish from the flow due to a gaze rotation around the vertical axis. Therefore, in the case of lateral translation, we would expect a clear contribution of peripheral vision to flow parsing.

In summary, the experiments reported here show a clear contribution of peripheral visual motion to the flow parsing process and further bolsters the evidence that this mechanism relies upon global optic flow fields.

## Supplementary Material

Supplementary material

## Author Note

Data and code is available from the ReShare research data repository, https://dx.doi.org/10.5255/UKDA-SN-852880.

## Note

1 Although this transformation is appropriate before combination of the different target trajectories, note that repeating the statistical analyses undertaken on the raw data does not change the conclusions.
