# Supplementary material for: Peripheral Visual Cues Contribute to the Perception of Object Movement During Self-Movement
Source: Iperception. 2017 Nov 21;8(6):2041669517736072. doi: 10.1177/2041669517736072 (PMC5700793; doi:10.1177/2041669517736072)
Supplement: Supplementary material [file supplementary_material.pdf]

Supplementary Materials

Methodological Details: M-Scaling

As the cortical representation of the retina is not uniform or equal across the visual field (Anstis, 1998) a fixed width flow stimulus presented in central vision would have been over represented in terms of the neural area activated by the stimulus in comparison to the same stimulus presented in peripheral vision (Mora et al., 1989). The cortical magnification factor provides a measure of the number of millimetres on the cortex that equates to one degree of visual angle on the retina (Anstis, 1998). This measure can be used to equate visual stimuli for neural area such that peripheral stimuli are scaled according to the reciprocal of the magnification factor (M-scaling).

Table S1 – Screen dimensions and angular equivalents of flow stimulus for each eccentricity condition. M-scaling factor and scaled width of the flow annulus are shown.

Visual angle of radius (degrees)	Inner radius of flow annulus (cm)	M-Scaling factor	Width of flow annulus (cm)	Width of flow annulus (degrees)
22.78	16.8	1	10.00	14.04
26.57	20	1.14	11.43	15.95
32.00	25	1.36	13.29	18.38
36.87	30	1.53	15.33	20.97
41.19	35	1.70	16.96	22.98

Full statistical results of Experiment 1

Table B1 –Detailed results of within-subjects ANOVA of Experiment 1 showing significant interactions at each flow eccentricity.

Flow eccentricity	ANOVA result comparing relative tilt at each target position
16.5 cm radius	$F(1, 5) = 118.643, p < 0.01$
20 cm radius	$F(1, 5) = 366.069, p < 0.01$
25 cm radius	$F(1, 5) = 56.967, p < 0.01$
30 cm radius	$F(1, 5) = 232.495, p < 0.01$
35 cm radius	$F(1, 5) = 130.394, p < 0.01$

Follow-on Experiments

The first follow-on experiment (Experiment S1) examined the contribution of the horizontal and vertical periphery. A peripheral contribution to parsing was measured with horizontal, vertical, and full peripheral fields. The results showed approximately equal effects in the horizontal and vertical conditions and the magnitude of the effect in the full field condition was predicted by a linear combination of both separate flow regions. The second follow-on experiment (S2) explored whether the smaller contribution of the far periphery in comparison to the near periphery could be accounted for by underlying differences in sensitivity to motion. Speed discrimination thresholds were determined for the near and far peripheral flow stimuli. This experiment revealed no significant differences in motion perception between the two peripheral regions.

Experiment S1: radial location and flow parsing

In macaque MT the lower visual field is over represented in comparison to the upper visual field (Maunsell & van Essen, 1987), and this overrepresentation has also been suggested in the peripheral visual field (Naito, Kaneoke, Osaka & Kakigi, 2000). Similar asymmetries have also been demonstrated in humans (see Skarandies, 1987 for a

review), suggesting that the lower visual field is especially suited to motion perception. In this experiment we examine whether the contribution to flow parsing of peripheral vision differs as a function of radial location. In Experiment 2 we presented visual flow in the left and right periphery to simulate self-movement. However, if the lower (or upper) visual field is particularly important for flow parsing then we might have found a greater contribution of peripheral vision to flow parsing if we presented flow in these quadrants of the visual field.

To test this, the peripheral display was modified in order to present flow in designated quadrants of the visual field and assess whether magnitude of relative tilt varied as a function of the flow configuration. The flow was positioned at the top and bottom of the screen (above/below condition) the left/right or in both locations.

Methods

Participants.

Six participants (2 male) took part in all conditions. Individuals were undergraduate (N = 4) or postgraduate students (N = 2). The same ethical procedures and eligibility restrictions used in Experiment 1 were employed. Aside from the author (CR), all other participants were naïve as to the experimental hypotheses.

Apparatus and stimuli.

Apparatus was the same as in Experiment 1. The Near peripheral stimulus used in Experiment 1 was modified; two 90 degree black segments (separated by 90 degrees) obscured two quadrants of the flow field in the Above/Below and Left/Right conditions (Figure S1). In addition to the two partial flow conditions, a full flow field condition was

also presented in order to compare the magnitude of the peripheral contribution to flow parsing in the partial conditions to that observed when flow area was not restricted.

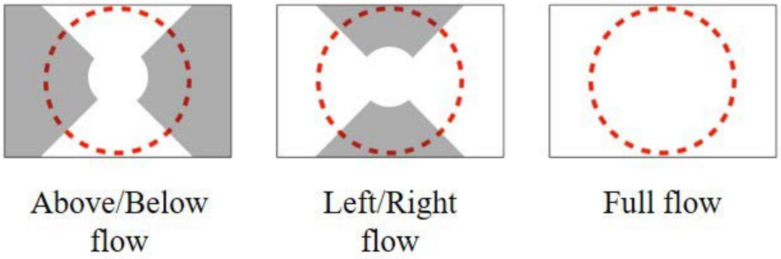

Figure S1 - Schematic diagrams for each flow configuration. Grey areas indicate the excluded flow regions. The central region was left unmasked so that the target and fixation point remained visible.

The target had a diameter of 0.2cm (0.12 degrees and it was presented 4 degrees from fixation. In a change from the previous experiment, the starting position of the target was shifted away from directly above/below fixation to either -45 degrees or +135 degrees (where 0 degrees is defined as vertically upwards from fixation, +CW). Targets were positioned at these locations so that, regardless of flow configuration, they were equidistant from the flow stimulus in all conditions.

Design.

Three IVs were manipulated: Flow direction (Expanding, Contracting), Target position (-4/+4 degrees), and Flow configuration (Above/Below, Left/Right, Full Flow). For each condition, 17 different target trajectories were presented from a range of ±16 degrees in 2 degree steps. Each flow configuration condition was run in a separate experimental block of 68 trials, in which the order of conditions (Flow direction, Target position and target trajectory) was randomised. The DV was relative tilt in degrees. A within-subjects design was employed and the order in which participants completed the

flow configuration blocks was counterbalanced across observers. Each observer completed all blocks within a single experimental session.

Procedure.

Procedure, trial timings, target speed and duration, flow speed and response format were the same as in Experiment 1. The response line was located in the same position as the target, which in this experiment was either -45 or +135 with respect to the fixation dot. One enforced break of 15 seconds occurred in each experimental block.

Results and discussion

The three flow configurations all showed the expected interaction between flow direction and target position (F (1, 5) = 33.800, p = 0.002), indicating a peripheral contribution to flow parsing. A 3 (Flow configuration) x 2 (Flow direction) x 2 (Target position) repeated measures ANOVA revealed that there were differences in this relationship between the flow configuration conditions in a three-way interaction (F (2, 10) = 18.567, p < 0.001).

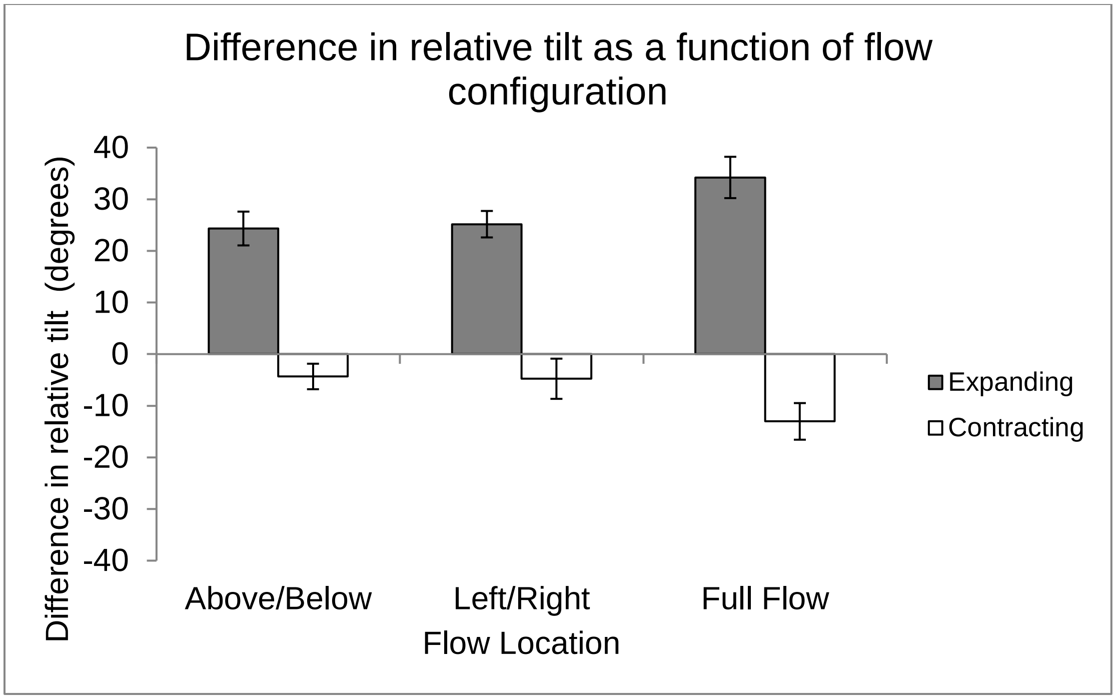

Figure S2 - Difference in relative tilt as a function of flow configuration and flow direction. Error bars show within-subjects *SE*.

In all three conditions the Expanding condition produced a positive tilt difference, and the contracting condition, a negative tilt difference (Figure S2); in line with a peripheral contribution to flow parsing. As before, the unsigned difference in relative tilt was much larger in the Expanding condition compared to the Contracting condition (F (1, 5) = 27.884, p = 0.003).

In the case of Contracting flow, it appears that the magnitude of relative tilt observed in the two partial flow conditions sums to approximately the same as the Full Flow condition (Figure S2). To further investigate this a linear regression was conducted to assess whether the sum of the tilts left/right and above/below conditions predicted the data in the Full flow condition. The Full Flow relative tilt was predicted by the sum of the Above/Below and Left/Right relative tilts (β = 0.535) and explained 55% of the variance in the Full Flow data (R^2 = .546, F (1, 10) = 12.016, p = 0.006).

A linear regression was also conducted for the Expanding flow condition to test whether the Full Flow relative tilt was predicted by the sum of the Above/Below and Left/Right relative tilts. This analysis also revealed that the linear sum predicted the Full Flow data (β = 0.652) and as a predictor explained 93% of the variance in the Full Flow data (R^2 = .925, F (1, 10) = 123.262, $p < 0.001$).

These regression analyses both indicate that for expanding and contracting flow, or forward and backward self-movement, the upper and lower *and* left and right peripheral visual field contribute flow parsing and that contributions are sub-additive[1].

Comparisons were also drawn between the two partial flow configurations, to ascertain whether there were any differences as a function of flow location. Participants reported similar relative tilts for the left/right and above/below conditions and the ANOVA confirmed there was no main effect of flow location (F (2, 10) = 0.261, p = 0.776, *n. sig*) and the interaction between flow location and flow direction was also not significant (F (2, 10) = 1.319, p = 0.310, *n. sig*).

The lack of differences between the left/right and above/below conditions indicates that these retinal locations in the periphery equally contribute to flow parsing.

Experiment S2: Speed discrimination thresholds

Previous work has suggested that sensitivity to motion does not decline as stimuli move further into the periphery. Orban, Kennedy and Bullier (1986) have shown that

[1] When calculating the size of the reduction in tilt between Full Flow and Left/Right conditions, one participant's reduction was 2 standard deviations below the group mean in the contracting condition and was therefore removed from the Expanding and Contracting analysis.

thresholds for velocity discrimination are maintained with increasing retinal eccentricity. However, they also note that there are differences in the velocity cut-off (i.e. the maximum speed that can be discriminated), which is higher in peripheral vision than central vision.

Another potential contributor to the difference between the Near and Far results found in Experiment 2 is the difference in stimuli. Although the stimuli used in the present experimental work were designed to provide a robust cue to self-movement, it may be that the dots (near periphery) and stripes (far periphery) did not equally stimulate the visual system, with one cue providing a more robust cue to self-movement than the other.

In this experiment, visual motion sensitivity was assessed through the measurement of speed discrimination thresholds. A higher threshold for Far than Near peripheral flow might provide an explanation for the lower contribution of Far periphery to flow parsing. A lack of difference (or a reversed pattern) would point to a functional account of a reduced input for peripheral vision in flow parsing.

Alongside identifying if motion perception differs between the peripheral conditions, a secondary aim of this experiment was to assess whether there are differences in sensitivity to motion for expanding and contracting stimuli. In previous experiments, there were differences in the magnitude of the effect for expanding and contracting flow, which may be underpinned by differences in motion sensitivity to these flow stimuli in peripheral vision. One explanation for this difference is that observers are more sensitive to expanding flow than contracting flow. Given that we are more

frequently exposed to expanding flow than contracting flow in everyday life it may be the case that the perception of self-movement and the subsequent parsing process is more effective during forward than backward self-motion.

In the present experiment, the Near and Far peripheral stimuli from Experiment 2 were used but the target was omitted from the display. The methodology employed in this experiment was closely based on the speed discrimination task used by Snowden and Kavanagh (Experiment 3; 2006).

Methods

Participants.

Six postgraduate students (3 male) with an age range of 24 to 27 were recruited and received payment at a rate of £10/hour for their time. The author (CR) was a participant in this experiment but all other participants were naïve as to the experimental hypotheses. Participant restrictions and ethical procedures from Experiment 1 were applied.

Apparatus and stimuli.

Apparatus and the near and far peripheral flow stimuli were identical to those used in Experiment 2 (see Figure 3), except that no target was presented. In this experiment, forced choice judgements were required and participants responded using the left and right mouse buttons.

Design.

In separate experimental blocks two IVs were manipulated: Flow direction (2 levels: Expanding or Contracting) and Peripheral condition (3 levels:

Near/Far/Combined). Expanding and contracting stimuli were presented in alternating blocks to measure speed discrimination thresholds independently for expanding and contracting flow and allow for an independent assessment of speed perception in these two cases. Within each of these conditions, an adaptive staircase method was employed in order to manipulate the difference in speed between the test stimulus and the reference stimulus (see Staircase Design, below). The DV was the speed discrimination threshold. The method for calculating the speed discrimination threshold is given in the Analysis section, below. All participants took part in all conditions in a within-subjects design and the order of the conditions was counterbalanced across observers.

Procedure.

As in the previous experiment, participants were seated at a distance of 95 cm from the projection screen and fixated a centrally presented point during stimulus presentation. On each trial, two stimuli were presented which were always in the same peripheral region (i.e. near and near or far and far) and always displayed motion in the same direction (i.e. both expanding or both contracting). One stimulus was always the reference stimulus, which was set to the same forward/backward translation speed used in previous experiments (30 cm/s). On each trial the reference stimulus was randomly presented in either the 1st or the 2nd stimulus interval. Participants indicated whether they perceived the stimulus as moving faster in the first or second interval.

The timeline of each trial closely followed those used in the preceding experiments. Once the participant clicked the mouse to start the trial, a fixation dot was presented for 1.2 seconds prior to the onset of the first stimulus. The first flow stimulus was presented for 2 seconds, followed by an Inter Stimulus Interval (ISI) of 0.5 seconds

during which a black screen was displayed. This was immediately followed by the second stimulus which was presented for 2 seconds (the same duration as the simultaneous flow and target presentation in the other experiments). Following the presentation of the two flow stimuli, a black screen was then presented until the observer responded. Following a button press, a white screen was displayed for 1.5 seconds before the start of the next trial. This short break was included to provide a break for participants in order to minimise the build-up of motion after effects across trials and prevent dark adaptation. Introducing this delay between trials stopped the participant from constantly being exposed to the motion stimulus, and therefore reduced the possibility of interaction between the stimuli on adjacent trials.

Staircase design.

The data was collected using two interleaved staircases (Levitt, 1971), one 3-up, 1-down and 1-up, 3-down rule. These staircases converge on the 21% and 79% point on the psychometric function. The data was subsequently used to reconstruct the psychometric function (see Analysis section).

At the start of each experimental block, the 3-up, 1-down staircase began with a test speed of 5 cm/s and the 1-up, 3-down staircase began with a test speed of 55 cm/s. Following a correct response in both cases, the speed of the test stimulus was thereafter selected by the staircase procedure from within a constrained range of approximately ±40% of the reference speed (17.5 cm/s to 42.5 cm/s). Thus, after the initial correct response, the difference between the reference (30 cm/s) and test stimulus, was never more than 12.5 cm/s. Step-size began at 10 cm/s and following the first reversal reduced to 5 cm/s and then to 2.5 cm/s following the second reversal. Thus, the minimum possible

difference between the test speed and the reference speed was 2.5 cm/s. The staircase terminated once 10 reversals of each staircase had been completed.

Analysis.

The psychometric function for each participant in each condition was reconstructed using the staircase data. The data was fitted using a Gaussian cumulative distribution function in Matlab (Żychaluk & Foster, 2009).

The test speeds on the psychometric function that equated to the respondent judging the test stimulus to be faster than the reference 75% of the time and 25% of the time were converted to the difference in speed by subtracting the reference speed (30 cm/s) from each value. The absolute difference in speed was then averaged across these two data points to provide a single measure of the minimum difference in speed that could be discriminated. This speed discrimination threshold was then used for within-subject comparison across conditions.

A 2 (Near/Far) x 2 (Expanding/Contracting) within-subjects ANOVA was conducted to assess whether speed discrimination thresholds differed between the near and far periphery or between expanding and contracting flow conditions.

Results and discussion

There were no significant differences in speed discrimination thresholds between the near and far peripheral flow conditions (F (1, 5) = 2.659, p = 0.164; see Figure S3).

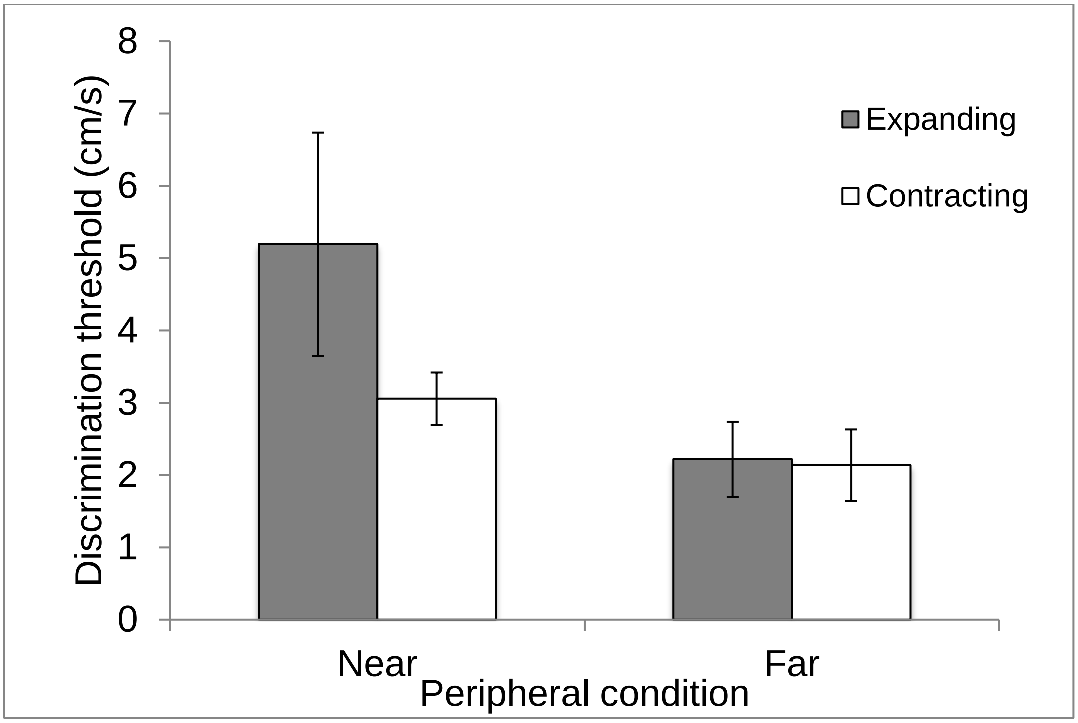

Figure S3 - Speed discrimination threshold (cm/s) as a function of peripheral flow condition and flow direction. Error bars show within-subject *SE*.

There were no differences between the speed discrimination thresholds for the two flow directions (F (1, 5) = 2.131, p = 0.204, *n. sig*) and there was also no interaction between flow direction and peripheral flow condition (near/far) (F (1, 5) = 1.407, p = 0.289, *n. sig*). Given the nature of these tests and the dependence on a null result we also conducted a Bayesian t-test analysis of the data to compute a scaled Jeffrey-Zellner-Siow Bayes factor using the method described by Rouder, Speckman, Sun, Morey and Iverson (2009, with the default prior, see http://pcl.missouri.edu/bf-one-sample). This analysis supported the null hypothesis that there was no difference between the forward and backward flow direction conditions and indicated that the null hypothesis was 1.75 times more likely than the alternative hypothesis. Similarly, the null hypothesis that there was no difference between the peripheral flow region conditions was 2.18 times more likely than the alternative hypothesis. The lack of significant differences in speed discrimination

between the four experimental conditions suggests that motion perception is equivalent between the two peripheral displays; regardless of the direction of self-motion.

Raw data and analysis files

Raw data, analysis code, and statistical outputs are stored in an online notebook and access can be requested from the corresponding author.
